# Implementing mHealth-Enabled Integrated Care for Complex Chronic Patients With Osteoarthritis Undergoing Primary Hip or Knee Arthroplasty: Prospective, Two-Arm, Parallel Trial

**DOI:** 10.2196/28320

**Published:** 2021-09-02

**Authors:** Jordi Colomina, Reis Drudis, Montserrat Torra, Francesc Pallisó, Mireia Massip, Eloisa Vargiu, Nuria Nadal, Araceli Fuentes, Marta Ortega Bravo, Felip Miralles, Ferran Barbé, Gerard Torres, Jordi de Batlle, The CONNECARE-Lleida Group

**Affiliations:** 1 Servei de Cirurgia Ortopèdica i Traumatologia Hospital Universitari de Santa Maria de Lleida Universitat de Lleida Lleida Spain; 2 Servei Anestesiologia Reanimació i Clínica del Dolor Hospital Universitari de Santa Maria de Lleida Universitat de Lleida Lleida Spain; 3 Group of Translational Research in Respiratory Medicine Institut de Recerca Biomedica de Lleida (IRBLleida) Lleida Spain; 4 eHealth Unit Eurecat Centre Tecnòlogic de Catalunya Barcelona Spain; 5 Gerència Territorial de Barcelona, Institut Català de la Salut Barcelona Spain; 6 Atenció Primària Àmbit Lleida, Institut Català de la Salut Lleida Spain; 7 Research Support Unit Lleida Fundació Institut Universitari per a la recerca a l'Atenció Primària de Salut Jordi Gol i Gurina (IDIAPJGol) Lleida Spain; 8 Centre d'Atenció Primària Cappont, Gerència Territorial de Lleida Institut Català de la Salut Barcelona Spain; 9 Universitat de Lleida Lleida Spain; 10 Center for Biomedical Network Research in Respiratory Diseases (CIBERES) Madrid Spain; 11 see Authors’ Contributions

**Keywords:** mHealth, osteoarthritis, arthroplasty, health plan implementation, chronic disease, mobile phone

## Abstract

**Background:**

Osteoarthritis is a disabling condition that is often associated with other comorbidities. Total hip or knee arthroplasty is an effective surgical treatment for osteoarthritis when indicated, but comorbidities can impair their results by increasing complications and social and economic costs. Integrated care (IC) models supported by eHealth can increase efficiency through defragmentation of care and promote patient-centeredness.

**Objective:**

This study aims to assess the effectiveness and cost-effectiveness of implementing a mobile health (mHealth)–enabled IC model for complex chronic patients undergoing primary total hip or knee arthroplasty.

**Methods:**

As part of the Horizon 2020 Personalized Connected Care for Complex Chronic Patients (CONNECARE) project, a prospective, pragmatic, two-arm, parallel implementation trial was conducted in the rural region of Lleida, Catalonia, Spain. For 3 months, complex chronic patients undergoing total hip or knee arthroplasty and their caregivers received the combined benefits of the CONNECARE organizational IC model and the eHealth platform supporting it, consisting of a patient self-management app, a set of integrated sensors, and a web-based platform connecting professionals from different settings, or usual care (UC). We assessed changes in health status (12-item short-form survey [SF-12]), unplanned visits and admissions during a 6-month follow-up, and the incremental cost-effectiveness ratio.

**Results:**

A total of 29 patients were recruited for the mHealth-enabled IC arm, and 30 patients were recruited for the UC arm. Both groups were statistically comparable for baseline characteristics, such as age; sex; type of arthroplasty; and Charlson index, American Society of Anesthesiologists classification, Barthel index, Hospital Anxiety and Depression scale, Western Ontario and McMaster Universities Osteoarthritis Index, and Pfeiffer mental status questionnaire scores. Patients in both groups had significant increases in the SF-12 physical domain and total SF-12 score, but differences in differences between the groups were not statistically significant. IC patients had 50% fewer unplanned visits (*P*=.006). Only 1 hospital admission was recorded during the follow-up (UC arm). The IC program generated savings in different cost scenarios, and the incremental cost-effectiveness ratio demonstrated cost-effectiveness.

**Conclusions:**

Chronic patients undergoing hip or knee arthroplasty can benefit from the implementation of patient-centered mHealth-enabled IC models aimed at empowering patients and facilitating transitions from specialized hospital care to primary care. Such models can reduce unplanned contacts with the health system and reduce overall health costs, proving to be cost-effective. Overall, our findings support the notion of system-wide cross-organizational care pathways supported by mHealth as a successful way to implement IC for patients undergoing elective surgery.

## Introduction

The progressive aging of populations has led to an increased burden of chronic diseases [1]. Osteoarthritis (OA) is one of the most disabling chronic diseases in developed countries. Worldwide estimates show that 10% of men and 18% of women aged >60 years have symptomatic OA [2]. Knees and hip are the most affected locations. Among the people with OA, 80% have movement limitations and 25% are disabled to perform major activities of daily living [2]. OA is associated with increased comorbidities and mortality. More than half of people with OA have another chronic medical condition, and approximately one-third of people have five or more chronic conditions [3].

Total hip arthroplasty (THA) and total knee arthroplasty (TKA) are effective surgical treatments for end-stage OA, improving joint function and health-related quality of life (QoL) [4]. Since 2000, the number of hip and knee replacements has increased rapidly in most countries in the Organization for Economic Co-operation and Development [5]. On average, hip replacement rates increased by 30% between 2007 and 2017, and knee replacement rates increased by 40% [5]. Comorbidities are independent predictors of increased postoperative complications and nonhomebound discharge in patients undergoing shoulder, hip, or knee arthroplasty [6] and are associated with physical function and pain after primary TKA [6].

So far, the strategies aiming to improve the outcomes of elective surgeries have mainly focused on enhanced recovery protocols, prehabilitation, and postoperative rehabilitation protocols, which have been proven effective for lower limb arthroplasty [7,8]. Nevertheless, there are aspects of the entire care process that can still be improved. Traditional care models suffer from care fragmentation, with the different care levels failing to communicate effectively. After transitioning from hospital care to primary care, patients can have changes in prescribed drugs or repetition of tests because of a lack of communication across health settings [9]. Moreover, traditional models still focus on diseases rather than patients, which makes patients and their caregivers passive actors in the process [9]. Therefore, there is a need for a profound redesign of how care is provided to chronic older patients to ensure quality and sustainability [10]. Integrated care (IC) models aim to increase health efficiency through defragmentation of care, including the promotion of collaboration across settings, promotion of patient-centeredness, and prioritization of preventive strategies [11]. Interestingly, the use of eHealth tools could be the key to enabling such models [12], as demonstrated by projects such as Personalized Connected Care for Complex Chronic Patients (CONNECARE) [13] or BeyondSilos [14], which have proven the feasibility of eHealth-enabled IC in the Catalan setting. The adoption of efficient IC models can result in better outcomes for THA and TKA. In this regard, a 2017 systematic review on the benefits of telerehabilitation after orthopedic surgery showed promising results for patients undergoing THA or TKA [15]. More recently, Jonker et al [16] confirmed the feasibility of perioperative eHealth interventions for the management of older surgical patients. However, there are very few initiatives that fully embrace the use of eHealth-enabled IC models in older patients undergoing THA or TKA.

The CONNECARE project is a European Union Horizon 2020 Research and Innovation project aiming to co-design, develop, deploy, and evaluate a novel smart and adaptive organizational IC model for complex chronic patients (CCPs) [17]. From 2016 to 2019, the project co-designed an organizational model for IC and an eHealth platform supporting it by means of an iterative patient-centered process involving patients and stakeholders across different health settings. The resulting IC model promoted collaboration among professionals in different care settings (hospital specialists, family physicians, and social workers), prioritizing home-based prevention strategies over institutional reactive care and fostering patient empowerment. A Smart Adaptive Case Management (SACM) system is offered as a web-based platform to professionals from different settings, and a patient-empowering mobile health (mHealth) self-management system allowing for three-level monitoring features and advanced communication is offered to patients.

As part of the CONNECARE project, a novel mHealth-enabled IC model was implemented in Lleida, Spain, targeting older CCPs undergoing elective THA or TKA. The existing care model for THA and TKA in Lleida is an enhanced recovery after surgery (ERAS) pathway based on different interventions to reduce perioperative stress; maintain and support homeostasis and physiological function; and accelerate the achievement of discharge criteria, including minimizing complications and readmission [18,19]. Although it produces good results with low transfusion, low complications, and decreased length of stay, it is limited by the scarce communication between professionals in different care settings, mostly hospital and primary care professionals, with different electronic medical records (EMRs) systems in hospitals and primary care centers (Argos SAP and ECAP [20], respectively). Therefore, we hypothesized that the new mHealth-enabled IC model could result in better outcomes for our patients.

This paper describes the results in terms of effectiveness and cost-effectiveness of the implementation of an mHealth-enabled IC model for the prevention of hospital readmissions in CCPs undergoing THA and TKA.

## Methods

### Study Design

This was a prospective, pragmatic, two-arm, parallel implementation trial comparing usual care (UC) with a 3-month mHealth-enabled IC intervention. The study was conducted from July 2018 to August 2019 at the University Hospital of Santa Maria (Lleida, Spain) and its network of primary care centers. This corresponds to a large rural area accounting for more than 236,000 citizens with a life expectancy of 80 and 86 years for men and women, respectively [21].

### Target Population

The eligibility criteria were home-dwelling patients elected for primary THA or TKA at the University Hospital of Santa Maria; aged >65 years; being defined as CCP (Charlson index score ≥3, taking four or more pills per day, and having had contact with the health system at least two times in the last 12 months); being classified according to the American Society of Anesthesiologists (ASA) classification as ASA II (mild systemic disease) or ASA III (severe systemic disease); and successfully passing a basic technological test, aimed to ensure the availability of internet connection at home as well as patients’ or caregivers’ competence with the use of a smartphone, tablet, or computer. The basic technological test can be found in Multimedia Appendix 1 [20,22].

### Recruitment

The recruitment was done in several waves to match the pace of the CONNECARE project technological developments. In each wave, consecutive potential participants were contacted by a case manager during preoperative assessment at the anesthesiology outpatient clinic. The case manager explained the study protocol and obtained informed consent. These patients formed the intervention arm. After the recruitment of each patient included in the intervention arm, an active search for a control with similar characteristics from the surgery waitlist of the Orthopedics Department of University Hospital of Santa Maria began. This enhanced the similarity of patients in the intervention and control arms, although it implied a certain lag in the recruitment of controls (from some days to few weeks). All patients and their caregivers, regardless of study arm, received a face-to-face explanation about the study and provided informed consent.

### Intervention

Patients in the intervention arm were attended using an IC model, including (1) preliminary assessment of the patient's health status using several questionnaires, tests, and indices specific to their main chronic diseases and social needs; (2) a self-management app, with status and performance reports, a virtual coach with customizable automated feedback, and full communication with the care team; (3) a Fitbit Flex 2 digital activity tracker [23], fully integrated into the self-management app; (4) a patient’s profile in the SACM web-based platform, accessible to all members of the care team (family physicians, hospital surgical team, and social workers) and used to coordinate professionals in the different settings and enable a communication channel among them and with the patient, when needed; and (5) assignment of a case manager in charge of supervising the whole process and being the main patient contact point. Additional details on the CONNECARE IC model and the supporting eHealth platform can be found in Multimedia Appendix 1. Patients in the control arm were attended using care as usual, being managed from primary care after hospital discharge. After discharge from the initial 90 days of UC or IC management, all patients had 3 months of additional passive follow-up.

### Data Collection

Variables characterizing the patients were collected at recruitment using the SACM on tablet and/or desktop computers. Collected variables included main baseline characteristics, such as age, sex, main chronic diseases, Charlson index of comorbidities [24], ASA scores [25], Barthel index for activities of daily living [26], Hospital Anxiety and Depression scale [27], assessment of the dwelling characteristics, main medications, Pfeiffer mental status questionnaire [28], and tobacco and alcohol consumption.

The cost estimation of the IC program and used health care resources is described in Multimedia Appendix 1. Briefly, IC costs included the costs of newly required medical personnel (hospital-based nurse case managers) and the costs of licensing and running the IC platform. In contrast, the one-off costs associated with the development of the IC model and supporting technology or the tuning of the system were not considered. Similarly, the required restructuring of health care professionals’ time to include new tasks related to the IC model was assumed to be covered by the health system at no additional cost, as we assumed that such restructuring would not imply any additional time. Costs associated with the use of health care resources, such as medical visits and hospital admissions, were based on official data from the Catalan Health Department [22]. All costs were originally in Euros (€) and were converted into US $ (conversion used: €1=US $1.21).

The main outcomes were (1) intervention effectiveness, as measured by the changes in the 12-item short-form survey (SF-12) health questionnaire’s physical and mental domains (baseline vs discharge) [29]; (2) use of health care resources after 6 months; and (3) cost-effectiveness, based on the improvement in QoL relative to costs, assessed by means of the incremental cost-effectiveness ratio (ICER). The source of health information was the EMR, and the collected information included hospital admissions, emergency room visits, visits to primary care, and visits to hospital specialists.

### Statistical Analyses

Participants’ baseline characteristics were described by n (%), mean (SD), or median (P25-P75), as appropriate. Comparisons between IC and control patients’ baseline characteristics were performed using the chi-square test, *t* test, or Kruskal-Wallis test, as appropriate. A paired *t* test comparing baseline with discharge was used to assess changes in the SF-12 domains. Linear regression models were used to assess the differences in the changes experienced by IC and control patients. Negative binomial regression models were used to assess the differences in the number of visits and admissions. Models were adjusted for age, sex, and Charlson score. ICER was calculated in relation to the SF-12 total score. Sensitivity analyses assuming two different scenarios, 150% and 200% estimated cost of the IC program, were performed to explore the cost-effectiveness performance of the IC model under unplanned increases in the implementation costs. Data analyses were conducted using Stata version 12.1 (StataCorp). The threshold for significance was set at a *P* value of .05. All *t* tests were two-tailed.

### Ethical Considerations

This study was approved by the Ethics Committee of Hospital Arnau de Vilanova (CEIC-1685), and all patients provided written informed consent. All collected data were handled and stored in accordance with the current national and international legislation.

## Results

**Figure 1:**
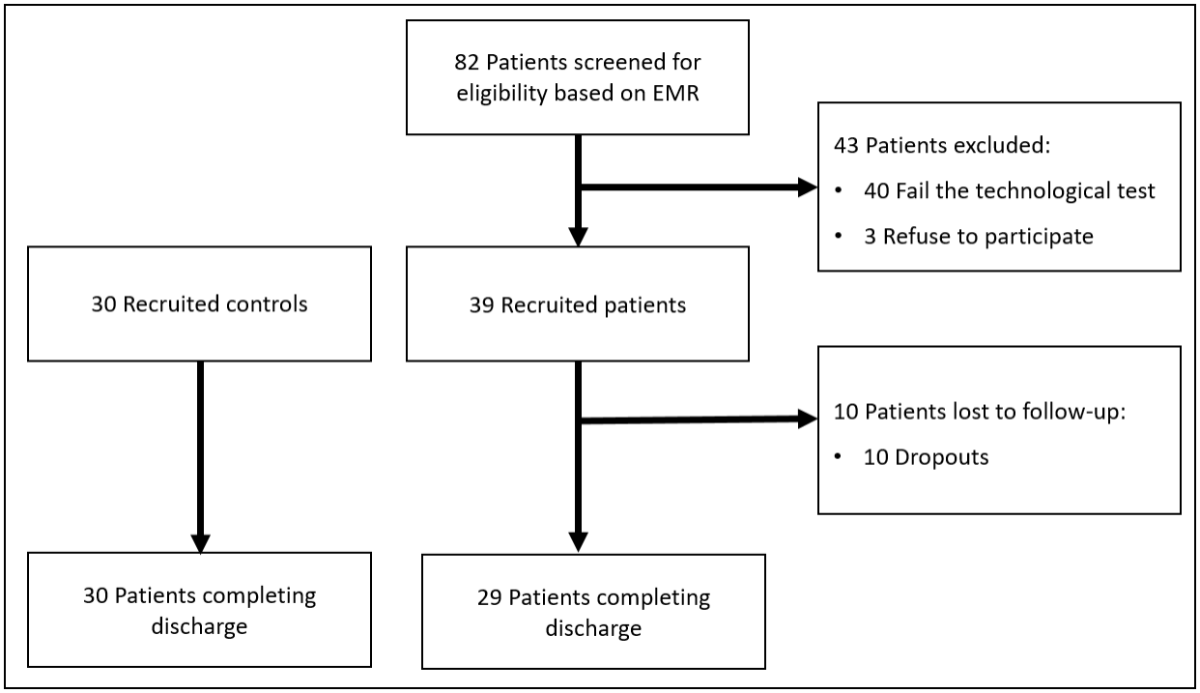
Study flowchart. EMR: electronic medical record.

Up to 82 patients were screened for eligibility. Of them, 49% (40/82) failed the technological test because they did not have an internet connection and 4% (3/82) refused to participate. Therefore, 39 patients were recruited for the mHealth-enabled IC arm and 30 for the UC arm. Final analyses were based on 29 IC and 30 control patients who completed the follow-up (Figure 1).

The baseline characteristics of the patients are presented in Table 1. Although the proportion of knee surgeries was slightly higher in the IC arm, no significant differences were found in the baseline characteristics of the patients in the two arms.

**Table 1:** Baseline characteristics of the patients in the usual care and integrated care arms (N=59).

Characteristic			Usual care (n=30)		Integrated care (n=29)		*P* value^a^	
Sex (male), n (%)			8 (27)		12 (41)		.23	
Age (years), mean (SD)			74 (8)		72 (9)		.46	
Charlson score, mean (SD)			4.3 (1.7)		4.2 (1.5)		.81	
**American Society of Anesthesiologists** **class, n (%)**								.52
	II	10 (33)		12 (41)				
	III	20 (67)		17 (59)				
**Western Ontario and McMaster Universities Osteoarthritis Index** **score, mean (SD)**								
	Pain	9.5 (3.4)		9.6 (3.8)		.93		
	Function	38.7 (12.8)		39.8 (14.2)		.77		
	Stiffness	2.3 (2.1)		3.0 (2.8)		.29		
Barthel score, median (IQR)			95 (90-100)		100 (95-100)		.16	
HAD^b^ scale anxiety score, mean (SD)			6.2 (4.9)		5.0 (3.9)		.34	
HAD scale depression score, mean (SD)			5.0 (2.3)		5.1 (2.9)		.88	
Pfeiffer intact intellectual functioning, n (%)			30 (100)		27 (93)		.14	
Surgery location: knee, n (%)			22 (73)		26 (90)		.11	

^a^Chi-square test, *t* test, or Kruskal-Wallis equality-of-populations rank test, as appropriate.

^b^HAD: Hospital Anxiety and Depression.

Table 2 shows the changes in QoL (SF-12 domains) from baseline to discharge. Regardless of the study arm, all patients showed a substantial increase in the SF-12 physical domain. The analysis of the differences in differences in QoL between IC and control patients showed no differences. Stratification according to the surgical location (hip or knee) reported similar findings.

**Table 2:** Changes in health status in the usual care and integrated care arms.

12-item short-form survey score		Baseline, mean (SD)	Discharge, mean (SD)	Change, mean (SD)	*P* value
**Physical**					
	UC^a^	27.9 (6.4)	42.0 (7.7)	14.1 (9.0)	<.001^b^
	IC^c^	29.9 (10.0)	45.3 (9.8)	15.4 (11.7)	<.001^b^
	Difference	2.0 (2.2)	3.3 (2.2)	1.4 (3.2)	.79^d^
**Mental**					
	UC	48.1 (14.2)	50.2 (13.5)	2.0 (11.9)	.35^b^
	IC	52.1 (14.2)	52.8 (12.9)	0.8 (15.2)	.79^b^
	Difference	3.9 (3.6)	2.6 (3.6)	−1.3 (5.1)	.94^d^
**Total**					
	UC	76.1 (15.6)	92.2 (18.1)	16.1 (14.8)	<.001^b^
	IC	81.9 (18.8)	98.1 (15.6)	16.2 (14.3)	<.001^b^
	Difference	5.9 (4.5)	6.0 (4.5)	0.1 (6.3)	.94^d^

^a^UC: usual care.

^b^Paired *t* test comparing baseline and discharge measures.

^c^IC: integrated care.

^d^Linear regression predicting the difference in the changes experienced by each arm, adjusted by age, sex, and Charlson index.

Table 3 shows that IC patients had 50% fewer unplanned visits than patients in the UC arm, either when considering visits directly related to the surgical procedure or any visits. One patient in the UC arm required hospital admission, whereas no admissions were recorded among IC arm patients.

**Table 3:** Total use of health services during the follow-up period (N=59).

Health service	Usual care (n=30), mean (SD)	Integrated care (n=29), mean (SD)	*P* value^a^	Adjusted *P* value^b^
All unplanned visits	1.4 (1.5)	0.7 (1.0)	.03	.006
Unplanned visits directly related to the surgery procedure	0.8 (1.2)	0.4 (0.7)	.10	.02
All hospital admissions	0.03 (0.2)	0 (0)	N/A^c^	N/A
Hospital admissions directly related to the surgery procedure	0.03 (0.2)	0 (0)	N/A	N/A

^a^Negative binomial regression model.

^b^Negative binomial regression model adjusted for age, sex, and Charlson comorbidity index.

^c^N/A: not applicable.

The analyses of within-trial costs and cost-effectiveness for all unplanned visits and hospital admissions are summarized in Table 4 and those for unplanned visits and hospital admissions related to the arthroplasty procedure are summarized in Table 5. The IC program generated savings from €109.88 (US $132.96) to €126.99 (US $153.66) per patient, depending on the nature of unplanned visits and hospitalizations. The IC program was cost-effective according to ICER, performing similar in terms of QoL gain while reducing overall expenses because of the reduction of unplanned visits and hospital admissions.

**Table 4:** Changes in health-related quality of life, within-trial costs (average cost per patient), and cost-effectiveness considering all unplanned visits and hospital admissions.

Variables	Usual care (n=30)	Integrated care (n=29)	Difference	ICER^a^
Changes in the 12-item short-form survey score, mean (SD)	16.1 (14.8)	16.2 (14.3)	0.1 (6.3)	N/A^b^
Unplanned visits costs^c^ (US $)	106.07	51.74	−54.33	N/A
Hospital admissions costs^c^ (US $)	185.25	0	−185.25	N/A
Total medical costs per patient (US $)	291.32	51.74	−239.58	N/A
Personalized Connected Care for Complex Chronic Patients program cost (US $)	0	85.92	85.92	N/A
Total costs per patient (US $)	291.32	137.66	−153.66	−1920.73

^a^ICER: incremental cost-effectiveness ratio; incremental cost associated with one additional point gain in the 12-item short-form survey.

^b^N/A: not applicable.

^c^Costs based on the Catalan Institute of Health official pricing.

**Table 5:** Changes in health-related quality of life, within-trial costs (average cost per patient), and cost-effectiveness considering unplanned visits and hospital admissions related to the surgical intervention.

Variables	Usual care (n=30)	Integrated care (n=29)	Difference	ICER^a^
Changes in the 12-item short-form survey score, mean (SD)	16.1 (14.8)	16.2 (14.3)	0.1 (6.3)	N/A^b^
Unplanned visits costs^c^ (US $)	64.68	31.05	−33.63	N/A
Hospital admissions costs^c^ (US $)	185.25	0	−185.25	N/A
Total medical costs per patient (US $)	249.93	31.05	−218.88	N/A
Personalized Connected Care for Complex Chronic Patients program cost (US $)	0	85.92	85.92	N/A
Total costs per patient (US $)	249.93	116.97	−132.96	−1661.94

^a^ICER: incremental cost-effectiveness ratio; incremental cost associated with one additional point gain in the 12-item short-form survey.

^b^N/A: not applicable.

^c^Costs based on the Catalan Institute of Health official pricing.

Sensitivity analyses assuming two different cost scenarios, 150% and 200% estimated cost of the IC program, thus exploring cost-effectiveness under unplanned increases in the implementation costs, showed savings and cost-effectiveness, as shown in Tables S1 and S2 in Multimedia Appendix 1.

## Discussion

### Principal Findings

The prospective assessment of the implementation of an mHealth-enabled IC program for TKA and THA management showed a reduction in the number of unplanned contacts with the health system after the surgery; generated substantial savings for the health system, while not having any negative impact on QoL or clinical outcomes; and demonstrated cost-effectiveness.

### Strengths and Limitations

The implemented IC model had several strengths that must be highlighted. First, there was an effort to involve all the stakeholders from different organizations that would be actors in a large-scale deployment of the mHealth-enabled IC program since the very early stages. This is key, as the lack of cooperation among professionals, teams, and organizations is a recurrent barrier for effective IC implementation [11]. Second, informal caregivers played a role in the IC process, as close relatives of the patients can be their link with the health system. Moreover, informal caregivers can play a key role in facilitating the use of self-management apps in older patients. Third, the self-management app for patients (allowing active monitoring, communication with the care team, and a virtual coach with customizable automated feedback) enhanced doctor-patient relationships [30] and facilitated early detection of any problem in the surgical recovery process [31]. Fourth, the assessment and promotion of patients’ physical activity is an effective way of preventing any mobility impairment, which is found in one-third of people aged >65 years [32]. Finally, the region selected for the deployment of the IC program, a large rural area, was especially appropriate as their citizens can benefit the most from community-based IC initiatives that can avoid unnecessary travel to the hospital.

Regarding this study, several strengths and limitations should be noted. Among the strengths, we note the use of a prospective study design with a comparator arm; the use of objectively measured endpoints, such as visits and admissions, in contrast to patient-reported outcomes; and cost and cost-effectiveness assessments. Concerning the limitations, the technological platform supporting the implemented IC model showed substantial improvements throughout the implementation period. This implied that patients recruited near the end of the implementation study had a richer IC experience than those recruited at the very beginning. Similarly, this had an impact on health care professionals, who had to cope with a platform under development and not fully integrated with existing EMRs. Nevertheless, participating patients and professionals showed great acceptability of the IC model and setting [33]. Moreover, directly participating in a dynamic development and implementation process fostered professionals’ engagement and allowed them to propose changes and new features to be developed, which resulted in not a single professional dropping out of the study. Next, the limited number of patients involved in the study had an impact on statistical power. Nevertheless, the current number of participants sufficed to show a significant reduction in robust endpoints, such as unplanned visits and hospital admissions, and showed the cost-effectiveness of the IC program. Finally, only direct costs were considered, although the inclusion of indirect or societal costs would most likely favor the cost-effectiveness of the IC model.

### Comparison With Existing Literature

This study aimed to assess the impact of the implementation of an IC model in three domains: (1) patients’ QoL, (2) patients’ use of health services, and (3) health economics. Regarding the QoL domain, the IC model performed as good as the UC arm in the differences-in-differences analysis. This result is in line with the mixed results found in a 2017 review on the impact of IC interventions on QoL [34]. However, it is worth noting that the great increase in QoL obtained after a successful THA or TKA is likely to mask any minor increase in QoL caused by being managed in an IC model. Next, regarding the use of health services, patients in the IC model required 50% fewer unplanned visits after the surgery. This corresponds to the upper margin of benefits reported in reviews about IC interventions between 2000 and 2015, which reported significant reductions in hospital activity ranging from 15% to 50% [35]. These excellent results could be in line with the notion of system-wide cross-organizational care pathways as a successful way to implement IC, in contrast to smaller and narrow interventions [9]. Finally, regarding health economics, IC generated savings from €109.88 (US $132.96) to €126.99 (US $153.66) per patient and was deemed cost-effective. This is in line with reviews stating the potential cost-effectiveness of IC in the management of chronic diseases [36]. However, these savings are lower than the range of US $584-$1434 obtained when applying the same IC model to CCPs with chronic obstructive pulmonary disease or heart failure [13], who are more prone to experiencing unplanned visits and hospitalizations.

When specifically focusing on THA and TKA, previous studies have suggested the usefulness of the different potential components of an eHealth IC model, including telerehabilitation [15], care pathways [18,37], education [38], patient-centeredness [39], mHealth continuous monitoring [40,41], and cross-setting integration [42]. However, to our knowledge, this study is the first to include all these components in a single mHealth-enabled IC model. Overall, our results are similar to or better than those reported in these previous studies, suggesting a moderate additive effect of combining the reported interventions into a single mHealth-enabled IC model.

### Implications for Research and/or Practice

The World Health Organization has already stated the need for patient-centered IC models to satisfy the health needs of older populations with chronic diseases while keeping costs sustainable [29,43]. With this premise in mind, the CONNECARE project tackled the task of iteratively co-designing a mHealth-enabled IC model with the participation of all key stakeholders: patients; hospital-based surgeons, anesthesiologists, nurses, physiotherapists, and case managers; primary care physicians and nurses; social caregivers; and managers, technical staff, developers, and researchers. This multidisciplinary team envisioned a system-wide cross-organizational patient-centered care pathway, in line with the principles of the 2015 World Report on Ageing and Health [1]. The experience acquired during the co-design and testing process allows us to highlight some key features that future IC models for the management of older citizens undergoing THA or TKA should consider (1) a common cross-setting web-based platform is key for a successful coordination of care across settings and patient monitoring; (2) the habilitation of functional communication channels for the patients can be a key source of savings, as most savings are achieved through the avoidance of unplanned visits; and (3) involving informal caregivers, such as younger family members, can facilitate the adoption of mHealth tools, such as sensors and self-management apps, and make the overall user experience very satisfactory [44]. Moreover, it is key to ensure access to an internet connection at home, as this was the main criterion halting the participation of potential users.

### Conclusions

The implementation of a patient-centered mHealth-enabled IC model for the management of patients undergoing THA or TKA successfully empowered patients, effectively connected the different care settings involved, reduced unplanned contacts with the health system, reduced health costs, and was cost-effective. This supports the use of mHealth tools for the implementation of system-wide cross-organizational IC models.

## Appendix

Multimedia Appendix 1 Supplementary information.

## Abbreviations

ASA: American Society of Anesthesiologists
CCP: complex chronic patient
CONNECARE: Personalized Connected Care for Complex Chronic Patients
EMR: electronic medical record
ERAS: enhanced recovery after surgery
IC: integrated care
ICER: incremental cost-effectiveness ratio
mHealth: mobile health
OA: osteoarthritis
QoL: quality of life
SACM: smart adaptive case management
SF-12: 12-item short-form survey
THA: total hip arthroplasty
TKA: total knee arthroplasty
UC: usual care
